# Spontaneous trans-anal evisceration of small bowel: a case report

**DOI:** 10.1093/jscr/rjaa582

**Published:** 2021-03-18

**Authors:** Omar Lasheen

**Affiliations:** Department of General and Colorectal Surgery, Royal Oldham Hospital Pennine Acute Trust, Oldham OL1 2JH, UK; Department of General Surgery, Cairo University Hospitals, Cairo, Egypt

## Abstract

Trans-anal evisceration of small bowel is a rare surgical presentation. The first case ever reported was in 1827. The exact mechanism of how this develops remains poorly understood.

A 70-year-old lady presented with multiple small bowel loops eviscerated through the anus. Abdominal exploration was done. The bowel was carefully reduced. There was a longitudinal defect on the anterior wall of the rectum at the recto-sigmoid junction and a large mesenteric defect and thrombosed mesenteric vessels compromising blood supply to part of the bowel. Resection of 50 cm of ileum, jejunostomy and a mucous fistula were performed.

Several preexisting pathologies such as rectal prolapse can result in thinning out of the wall of the rectum. That combined with increased intra-abdominal pressure can explain trans-anal evisceration of the bowel. This condition is managed by surgical intervention. The operation will depend on the extent of viability, contamination and patient’s general condition.

## INTRODUCTION

Trans-anal evisceration of small bowel is a very rare surgical presentation. The first case ever reported was in the early 19th century, and up until 2007 only 65 have been recorded [1]. The exact mechanism of how this develops remains poorly understood. Yet in the majority of the cases recorded there was a perforation in the anterior wall of the rectum or sigmoid colon [2].

## CASE REPORT

**Figure 1:**
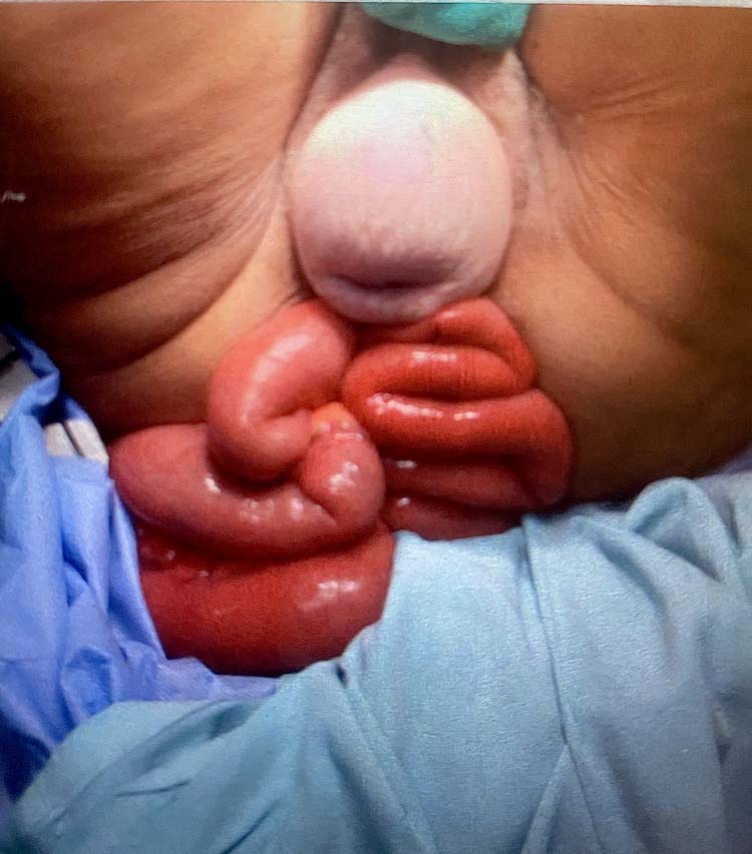
Multiple small bowel loops eviscerated through anus and associated long-standing uterine prolapse.

A 70-year-old, bed-bound female patient with a history of multiple strokes, presented to A + E with multiple loops of small bowel, eviscerated through the anus (Fig. 1). When asked, the family explained that the patient was chronically constipated and was on regular laxatives. She did not have any recent history of trauma. Her daughter had explained that she has been suffering from rectal prolapse which she could normally reduce with ease. However, this time what was prolapsing ‘from her back passage’ looked different, so she rushed her to the hospital.

The patient was hemodynamically stable. When examined, there was an associated uterine prolapse. The bowel loops were covered with warm saline soaked packs and the patient was immediately transferred to theater.

Abdominal exploration through an infra-umbilical incision was done; the bowel loops were carefully reduced through the anal canal. It appeared that there was a longitudinal defect on the anterior wall of the rectum at the recto-sigmoid junction. There was also a large mesenteric defect, possibly ensued from prolonged traction, and thrombosed mesenteric vessels compromising blood supply to part of the bowel, so resection of 50 cm of ileum with a jejunostomy and a mucous fistula were performed. The postoperative recovery was uneventful and the patient was discharged 16 days after surgery.

## DISCUSSION

We do not know for sure how many cases of small bowel evisceration have been reported. Brodie, in 1827, reported the first ever case. The exact mechanism remains unclear. A number of factors leading to increased abdominal pressure such as straining to defecate, vomiting, heavy lifting and labor can precipitate it [2, 3].

Excluding trauma, several preexisting surgical conditions have been suggested to preexist such as rectal prolapse, diverticular disease, uterine prolapse or solitary rectal ulcer all of which can result in thinning out of the wall of the rectum or sigmoid [4]. A large number of reported cases were elderly patients who had a history of recta prolapse [5]. One suggested mechanism that could explain development of this condition is that increased intra-abdominal pressure could cause a deepening of the rectovesical or rectouterine pouch, leading to invagination of the anterior rectal wall into the rectal lumen [3]. Another suggested mechanism is that with recurrent rectal prolapse there would be repeated friction resulting in fibrosis, development of varicosities and intramural bleeding with subsequent separation of the layers of the rectum particularly on the antimeseteric surface border _anterior wall_ of the rectum which has the least circulation [6].

Initial management involves adequate fluid resuscitation and reducing contamination by cleaning of the eviscerated bowels with normal saline. The treatment for this condition is typically surgical with laparotomy aiming at of reducing the small bowel, resection if indicated and repair of the rectal tear. A covering stoma is usually formed if deemed necessary, depending on bowel viability and overall state of contamination of the bowel [3, 5]. However, with advances in laparoscopic surgery, in 2005 Antony *et al*. [6] reported the first successful laparoscopic management of eviscerated small bowel repair of spontaneous rectosigmoid rupture.

## CONCLUSION

Several preexisting pathologies such as like diverticular disease, rectal prolapse, uterine prolapse or solitary rectal ulcer can result in thinning out of the wall of the rectum or sigmoid. That combined with increased intra-abdominal pressure can explain trans-anal evisceration of the bowel. This condition is typically managed by surgical intervention. The operation itself will depend on the extent of tissue contamination and viability along with the patient’s general condition.

## CONFLICT OF INTEREST STATEMENT

None declared.

## FUNDING

None.
